# Unusual Pheochromocytoma Presentation: From Dysuria to Catecholamine Crisis

**DOI:** 10.1210/jcemcr/luad059

**Published:** 2023-07-27

**Authors:** John Sun, Olga Senashova, Brian Brinkerhoff, James Lim

**Affiliations:** Division of Surgical Oncology, Department of Surgery, Oregon Health & Science University, Portland, OR 97239, USA; Department of Endocrinology, Oregon Health & Science University, Portland, OR 97239, USA; Department of Pathology and Laboratory Medicine, Oregon Health & Science University, Portland, OR 97239, USA; Division of Surgical Oncology, Department of Surgery, Oregon Health & Science University, Portland, OR 97239, USA

**Keywords:** pheochromocytoma, adrenal, catecholamine, hemorrhage

## Abstract

Pheochromocytomas are catecholamine-secreting tumors that can present as a surgical emergency, with a mortality rate as high as 15%. When these lesions present as a crisis, diagnosis and management can be very challenging, given the profound physiologic consequences, such as cardiovascular collapse or multiple organ failure, occurring over a rapid time frame. We describe an unusual case of a pheochromocytoma presenting with urinary frequency and subsequent shock and tumor hemorrhage following a urological procedure. Our patient was successfully managed with resuscitation and appropriate blood pressure control to stabilize hemodynamics prior to proceeding with open adrenalectomy. Furthermore, our patient presented initially with urinary symptoms, which has not been described as an initial presentation of pheochromocytoma. This case brings important learning points regarding unusually presenting pheochromocytomas and emergency management to improve patient survival.

## Introduction

Pheochromocytomas and paragangliomas are catecholamine-secreting neuroendocrine tumors that can have devastating hemodynamic implications. Pheochromocytomas primarily present with vague symptoms, including headache and high blood pressure unresponsive to multiple antihypertensive medications. However, these lesions can present with other rare symptoms that are resistant to medical management and eventually lead to interventions.

Our patient presented with dysuria. A urological workup concluded he had chronic cystitis and a spastic bladder. This workup culminated in a cystoscopy that provoked his catecholamine crisis. To the best of our knowledge, resistant and unexplained urinary symptoms have not been well reported in patients with adrenal pheochromocytomas. One study, in 1992, investigated urodynamics in pheochromocytoma patients, demonstrating that urinary retention could be explained by excessive alpha receptor stimulation at the bladder neck [1]. Furthermore, lower urinary tract symptoms are commonly described with bladder paragangliomas or as a result of metabolic disturbance such as glucosuria [2, 3]. Given the rare incidence of urinary symptoms, our patient's dysuria could also have been completely unrelated. Invasive procedures to further investigate the patient's complaints can lead to hemodynamic instability, due to either catecholamine excess or volume shifts, and instigate a crisis. The mortality rate due to pheochromocytoma crisis is described in the literature to be as high as 15% [4].

The definitive treatment for pheochromocytoma crisis is surgical resection; however, doing so in an elective or urgent setting as opposed to an emergency surgery has been shown to decrease mortality and morbidity [5, 6]. Emergent surgical intervention for pheochromocytomas has been shown to have mortality rate as high as 25% [7] Studies have demonstrated that patients presenting in a catecholamine crisis should be stabilized with alpha blockade and resuscitation before proceeding to the operating room for resection [4, 8]. We describe an unusual case of a hemorrhagic pheochromocytoma that initially presented with urologic symptoms that was managed nonoperatively until sufficient alpha and beta blockade was achieved to safely perform surgical excision.

## Case Presentation

A previously healthy, 45-year-old male, without a family history of adrenal tumors, developed hypertension, headaches, and frequent urination for 6 months prior to presentation. His blood pressure was initially managed with losartan, and he underwent evaluation by urology for dysuria. He was diagnosed with chronic cystitis and spastic bladder. After thorough workup, cystoscopy with bladder biopsies was performed in the operating room due to worsening of lower urinary tract symptoms. The procedure was uneventful, and he was discharged home. Later that day, he presented to the emergency department at a local hospital with worsening chest pain. Upon presentation, he was tachycardic to 160 beats per minute (bpm) and hypertensive to a systolic blood pressure of 220 mmHg. He was admitted to the intensive care unit (ICU) for suspicion of a myocardial infarction, acute pulmonary edema, and hypoxic respiratory failure. He underwent a computed tomography (CT) of chest, abdomen, and pelvis, which demonstrated a ruptured 9.7 × 8.7 cm adrenal mass with focal hyperdensity (30-55 Hounsfield units) suspicious for hemorrhagic component with hyperdense fluid extending superiorly and inferiorly in the left retroperitoneum from the posterior aspect of the mass.

About 8 hours after admission to the ICU, he developed cardiovascular collapse requiring maximal vasopressor support. Coronary syndrome was ruled out with a cardiac catheterization, but the patient developed acute renal failure requiring initiation of hemodialysis. Given his clinical presentation and CT scan findings, a pheochromocytoma was the presumed diagnosis and the patient started treatment with prazosin. Subsequently, renal function improved and hemodialysis was discontinued. The patient's blood pressure continued to be unstable and he developed another hypertensive event on hospital day 4 with associated worsening flank and abdominal pain. A CT angiogram was performed, which demonstrated new acute hemorrhage of the adrenal mass. At this point, the patient was then transferred to our institution.

## Treatment

**Figure 1.**
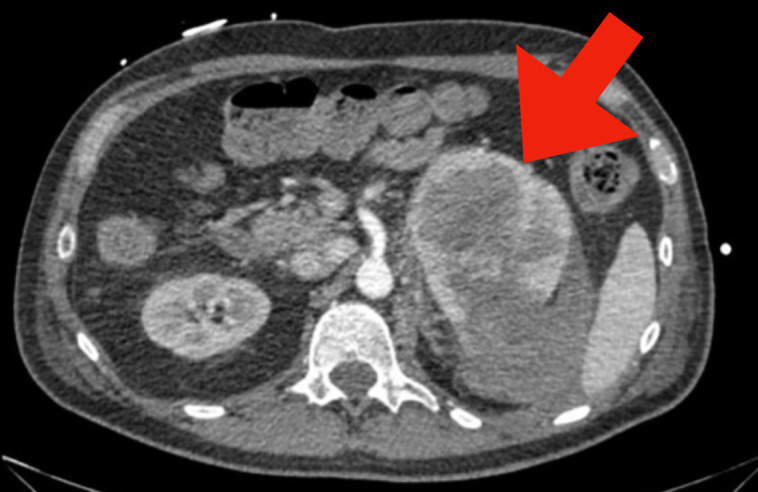
Axial CT angiogram demonstrating ruptured left adrenal mass hematoma with hyper dense blood products extending into the left retroperitoneum and pelvis but no active extravasation. The arrow identifies the lesion of interest. It is important to note the difference in density between the blood product and adrenal gland.

**Figure 2.**
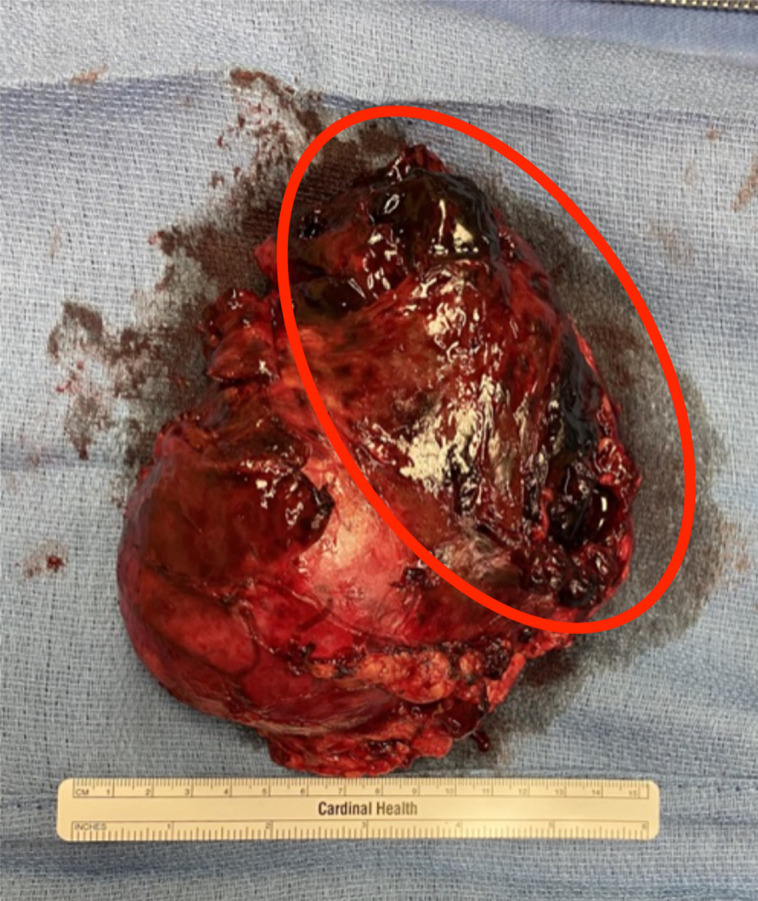
Photo of gross specimen from left adrenalectomy demonstrating hemorrhagic component of the pheochromocytoma (outlined in red). Specimen was removed in its entirety without violation of capsule.

**Figure 3.**
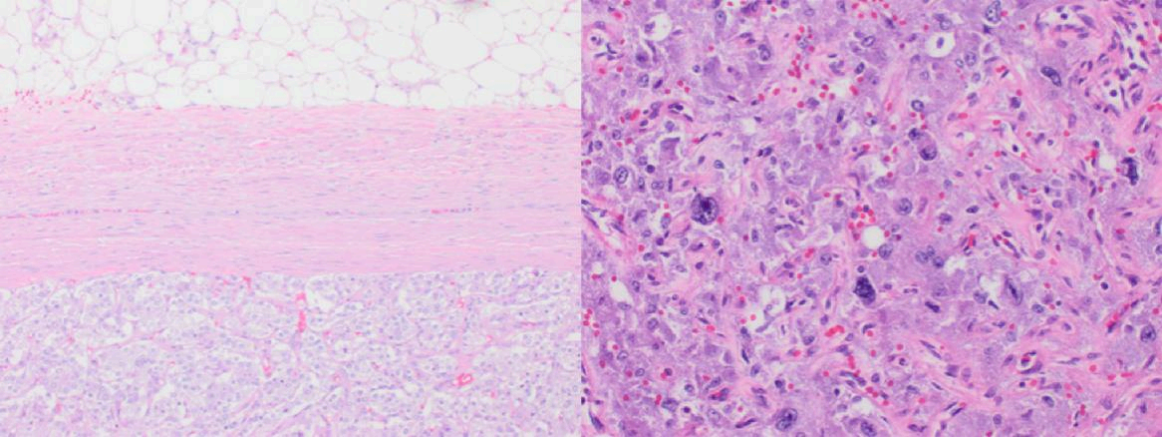
Final histologic evaluation. Left: pheochromocytoma abutting the capsule of adrenal gland demonstrating clear demarcation of tumor. Right: histology demonstrating large nests of tumor cells, high cellularity, cellular monotony, cell spindling, mitotic figures >3/10 HPF, and profound nuclear pleomorphism. PASS = 11 (>4 suggests potential malignant behavior) [9].

Upon arrival to our hospital, the patient was transfused with 2 units of packed red blood cells and was started on metyrosine and phenoxybenzamine. Biochemical workup confirmed a catecholamine-secreting tumor with plasma metanephrine of 17.9 nmol/L (0.65 mcg/dL) and normetanephrine >50 nmol/L (1.81 mcg/dL), and 24-hour urine metanephrines of 14 183 ug/24 hours (74 662 nmol/day) (Table 1). Once the patient's hemodynamic parameters stabilized, a CT angiography was obtained, which showed evidence of stable hemorrhage with no active extravasation (Fig. 1). The patient's hypertension and heart rate were controlled with a beta blocker (carvedilol 3.125 mg twice daily), nonselective and selective alpha receptor antagonists (phenoxybenzamine 30 mg 3 times daily, prazosin 4 mg every 6 hours), as well as metyrosine (500 mg every 6 hours) for 48 hours. Multidisciplinary discussion was held between all care teams and the patient, and the decision was made to proceed with resection during this admission. Fourteen days after transfer, an open left adrenalectomy was performed. His mass was quite vascular, and he had 2.5 L of blood loss intraoperatively but no hypertensive crisis (Fig. 2). Final pathology confirmed pheochromocytoma with Pheochromocytoma of the Adrenal Gland Scaled Score (PASS) of 11, with a score >4 concerning for a malignant tumor (Fig. 3) [9]. Germline genetic testing showed no pathogenic variants in genes that could predispose the patient to pheochromocytomas.

**Table 1.** Initial biochemical workup to confirm catecholamine-secreting tumor

Lab test	Conventional units	SI units	Reference range
Plasma metanephrine	17.9 nmol/L	0.65 mcg/dL	0-0.49 nmol/L 0-0.18 mcg/dL
Normetanephrine	>50 nmol/L	1.81 mcg/dL	0-0.89 nmol/L 0-0.03 mcg/dL
24-hour urine metanephrines	14 183 ug/day	74 662 nmol/day	55-320 ug/day 289.5-1684.5 nmol/day

## Outcome and Follow-up

He was discharged on postoperative day 5. At his 1-year follow-up assessment, he has recovered well and his blood pressure and urinary issues have all resolved. He has returned to his daily activities without issues. There has been no evidence of recurrence on imaging or biochemical testing.

## Discussion

Our case demonstrates a unique presentation of a pheochromocytoma in a patient with dysuria and subsequent shock and hemorrhage following a urologic procedure. Dysuria has not been a symptom frequently associated with adrenal catecholamine excess. These symptoms highlight the variable presentations of pheochromocytomas and should remain on the differential for these patients. Few reports have elucidated urinary symptoms as the initial presenting complaints in patients with pheochromocytoma. Urinary frequency has been described with paragangliomas of the bladder or in the pelvis [2]. Urodynamics of pheochromocytomas demonstrate a typical alpha-adrenergic overstimulation pattern and may explain bladder dysfunction as a presenting symptom [1]. Although it is possible that our patient's dysuria was unrelated to the pheochromocytoma, his urinary symptoms completely resolved after resection of his tumor. Regardless of association, further investigation of his urologic symptoms with invasive procedures likely triggered the patient's pheochromocytoma crisis. Delayed diagnosis of this unusually presenting tumor resulted in difficult-to-control hemodynamics with subsequent cardiac collapse; the patient's variable blood pressures during hospital course, prior to adrenalectomy, are likely the result of the hemorrhage. This illustrates the fragility of hemodynamics in these patients and highlights that even minor procedures can lead to cardiac collapse without appropriate medical preoptimization. In patients with known pheochromocytomas, any procedures should be performed under careful hemodynamic monitoring.

After transfer to our institution, we were able to manage the patient's hemorrhage without additional invasive procedures, after his blood pressure was stabilized on appropriate medications. He required adjuncts to the usual alpha blockade, such as metyrosine. Our multidisciplinary approach with endocrinology, intensivists, interventional radiology, and surgery allowed us to safely pursue a treatment paradigm that minimized morbidity.

The timing of surgery was discussed extensively during his admission. Although waiting several months to proceed with surgery may have led to an easier dissection and perhaps made a laparoscopic approach possible, the patient did not have access to a major hospital system at home and did not feel safe being discharged. Therefore, the decision was made to proceed with surgery during this admission. The surgery itself was challenging, as there was significant peri-adrenal stranding secondary to the hemorrhage, which made it difficult to identify the distinct borders of the adrenal mass. However, as it is standard practice during an adrenalectomy to resect the adrenal en bloc with peri-adrenal adipose tissue en bloc, this did not preclude a safe and oncologic resection (Fig. 2).

The incidence of hemorrhagic pheochromocytomas is rare and the optimal treatment remains difficult in these critically ill patients. Within the published literature, there are fewer than 70 cases that have been described [8, 10]. The ability to stabilize the hemorrhage likely determines the need for urgent intervention vs supportive care. Case reports that have had free intraperitoneal hemorrhage usually required urgent intervention with either embolization or surgery [7]. When the hemorrhage is contained within the retroperitoneum, it should be able to be managed with supportive care and surgery can be carried out in a more elective fashion. Emergent interventions in hemorrhagic pheochromocytomas carry a significantly higher risk of longer hospitalization, ICU admission, peri-operative complications, and death [5]. By being able to delay the surgery, patients can obtain adequate alpha blockade, allowing for better hemodynamics and tolerance for the operation. Our patient had a catastrophic hemodynamic collapse due to an unrecognized pheochromocytoma but ultimately had an optimal outcome because he was able to be managed along this treatment algorithm.

Lastly, while histologic scoring systems such as PASS may be helpful in predicting malignant pheochromocytomas, local invasion and distant metastases are the only findings that confirm malignancy [9]. Genetic testing is recommended in all patients with pheochromocytomas because up to 40% of patients harbor a genetic mutation. Genetic mutations can inform us of the higher likelihood of developing metastatic disease. Because all pheochromocytomas are thought to have malignant potential, annual surveillance is recommended with biochemical testing (clinical imaging, as indicated) indefinitely. At his 1-year follow-up, our patient did not have any recurrence or metastasis.

## Learning Points

- Pheochromocytomas can present with a variety of symptoms, including urinary complaints.
- Patients with pheochromocytomas have fragile hemodynamics and even minor procedures can trigger crisis and have catastrophic consequences.
- To minimize morbidity and mortality of surgical resection, hemorrhagic pheochromocytoma crisis should be managed nonoperatively until patients have been resuscitated and stabilized hemodynamically.
- Genetic testing and pathology can be helpful in detecting high risk tumors, but patients should still be surveyed annually with biochemical (plasma or urine metanephrines) testing indefinitely and imaging as clinically indicated.

## Contributors

All authors made individual contributions to authorship.

J.S.: conceptualization, data curation, writing—original draft preparation, reviewing, and editing.

O.S.: writing—reviewing and editing.

B.B.: data curation, writing—reviewing and editing.

J.L.: conceptualization, data curation, supervision, writing—reviewing and editing.

## Data Availability

Original data generated and analyzed during this study are included in this published article.

## Abbreviations

CT: computed tomography
ICU: intensive care unit

## References

[1] Carnaille BM, Rigot JM, Bailleul JP, Quievreux JL, Wemeau JL, Proye CA. Urodynamics in patients with pheochromocytoma: a peri-operative study of 10 cases. World J Surg. 1992;16(4):676‐679. 1357831 10.1007/BF02067353

[2] Beilan JA, Lawton A, Hajdenberg J, Rosser CJ. Pheochromocytoma of the urinary bladder: a systematic review of the contemporary literature. BMC Urol. 2013;13:22. 23627260 10.1186/1471-2490-13-22 PMC3654956

[3] Edelman ER, Stuenkel CA, Rutherford JD, Williams GH. Diabetic ketoacidosis associated with pheochromocytoma. Cleve Clin J Med. 1992;59(4):423‐427. 1525976 10.3949/ccjm.59.4.423

[4] Meijs AC, Snel M, Corssmit EPM. Pheochromocytoma/paraganglioma crisis: case series from a tertiary referral center for pheochromocytomas and paragangliomas. Hormones (Athens). 2021;20(2):395‐403. 33575936 10.1007/s42000-021-00274-6 PMC8110488

[5] Scholten A, Cisco RM, Vriens MR, et al Pheochromocytoma crisis is not a surgical emergency. J Clin Endocrinol Metab. 2013;98(2):581‐591. 23284003 10.1210/jc.2012-3020

[6] Marti JL, Millet J, Sosa JA, Roman SA, Carling T, Udelsman R. Spontaneous adrenal hemorrhage with associated masses: etiology and management in 6 cases and a review of 133 reported cases. World J Surg. 2012;36(1):75‐82. 22057755 10.1007/s00268-011-1338-6

[7] Hanna JS, Spencer PJ, Savopoulou C, Kwasnik E, Askari R. Spontaneous adrenal pheochromocytoma rupture complicated by intraperitoneal hemorrhage and shock. World J Emerg Surg. 2011;6(1):27. 21843357 10.1186/1749-7922-6-27 PMC3178469

[8] Pacak K. Preoperative management of the pheochromocytoma patient. J Clin Endocrinol Metab. 2007;92(11):4069‐4079. 17989126 10.1210/jc.2007-1720

[9] Thompson LD. Pheochromocytoma of the Adrenal gland Scaled Score (PASS) to separate benign from malignant neoplasms: a clinicopathologic and immunophenotypic study of 100 cases. Am J Surg Pathol. 2002;26(5):551‐566. 11979086 10.1097/00000478-200205000-00002

[10] Lambrecht A, Inglis JM, Young R. Abdominal pain with intra-adrenal bleeding as an initial presentation of pheochromocytoma. BMJ Case Rep. 2021;14(1):e237975. 10.1136/bcr-2020-237975 PMC7802696 33431458

